# Robustness of Joint Over Separate Models for Investigating Predictors of Blood Sugar Level and Time to First Remission Among Type I Diabetic Patients Under Treatment; a Retrospective Study Design

**DOI:** 10.1002/hsr2.72427

**Published:** 2026-04-24

**Authors:** Maru Zewdu Kassie, Awoke Seyoum Tegegne

**Affiliations:** ^1^ Department of Statistics, College of Natural and Computational Sciences Assosa University Assosa Ethiopia; ^2^ Department of Statistics, College of Science Bahir Dar University Bahir Dar Ethiopia

**Keywords:** blood sugar level, Cox proportional hazard model, Joint model, linear mixed model, time to first remission

## Abstract

**Background and Aims:**

Diabetes mellitus (DM) is a major public health problem that is responsible for morbidity and mortality. Blood sugar levels in DM patients fluctuate based on self‐care management, influencing survival biomarkers such as death, complications, and recovery. A joint modeling approach was used to evaluate the relationship between these biomarkers, including their longitudinal trajectories and the corresponding survival times. The study aimed to identify factors affecting longitudinal blood sugar level measurements and time to first remission in T1DM patients at Debre Tabor General Hospital, Northwest Ethiopia.

**Methods:**

A retrospective study was conducted on 217 randomly selected T1DM patients from January 2018 to January 2020. The linear mixed model for the longitudinal part, the Cox PH model for the survival part, and the joint model for their association were used. The Kaplan‐Meier survival estimate and Log‐Rank test were utilized to assess and compare the survival times.

**Results:**

In the current study, about 67.7% of the patients had their first remission, and the rest 32.3% were censored. The estimate of the unobserved association parameter (α) in the joint model was −1.7914 (*p* < 0.001), indicating a strong negative correlation between the two sub‐models. Older age [AHR = 0.9746, *p* < 0.001], male gender [AHR = 0.1706, *p* < 0.001], comorbidities [AHR = 0.0783, *p* < 0.001], family history of diabetes mellitus [AHR = −2.661, *p* = 0.008], and anemia [AHR = 2.1833, *p* = 0.02] were associated with increased blood sugar levels and delayed remission.

**Conclusion:**

The joint model outperforms the separate model in terms of variability, goodness of fit, and statistical significance. Future studies should use joint modeling for longitudinal and survival data analysis. Targeted interventions for risk factors like age, gender, comorbidities, family history, and anemia may enhance remission and glycemic control.

## 1 Introduction

Diabetes mellitus is a disease in which the body does not produce or respond properly to insulin [1]. Managing it is a lifelong challenge that demands consistent self‐care to maintain effective glycemic control [2, 3]. Most of the time, it is noted that type II diabetes mellitus (T2DM) is a highly prevalent and serious health problem in the world. However, type I diabetes mellitus (T1DM) has also increased rapidly with many complications [4, 5]. This problem is also critical in Ethiopia, specifically in the study area [6]. In Ethiopia, diabetic patients must travel long distances to obtain insulin and tablets since there is a shortage of health facilities [7]. Insulin is one of the most valuable anti‐diabetic medications that controls the patient's blood sugar level [1]. In Patients who have related diabetic complications and other diseases like AIDS, kidney disease, stroke, hypertension, and the like, the tendency to control their glycemic level is low [8].

Globally, the epidemiological landscape of diabetes is evolving rapidly. According to the International Diabetes Federation (IDF) 2021 report, over 500 million people worldwide were living with diabetes. This number is projected to increase to 578 million (10.2%) by 2030 and to 700 million (10.9%) by 2045 [9]. Notably, the prevalence of diabetes is expected to rise more sharply in high‐income countries, reaching approximately 10.4%, compared to 4.0% in low‐ and middle‐income countries. This indicates a disproportionately higher burden in developed nations; however, the growing trend in developing countries should not be underestimated. These comparisons must also be interpreted with caution, as diagnostic capacity and health system infrastructure differ significantly across regions. However, these figures may not fully capture the true burden in developing regions, where limited diagnostic capabilities and underreporting are common [10]. In many African countries, including Ethiopia, diabetes remains underdiagnosed and often goes unnoticed until complications arise, contributing to a growing public health challenge [6, 11].

An earlier study was conducted on determinants of the recovery time of diabetic patients, and some others focused on longitudinal blood sugar level control. US studies show that financial hardship, like bill payment struggles, impacts glycemic control [12]. Another study found that factors such as hypertension history, diet, age, education level, diastolic blood pressure, and time were significant for increased blood sugar levels [13]. Additionally, our prior research identified age, low hemoglobin levels, comorbidities, and family history of diabetes as key factors for elevated fasting blood sugar levels [14]. Furthermore, a study found that iron deficiency anemia is associated with higher HbA1c levels in T1DM patients with similar glycemia [15]. Another study found that T1DM patients had higher poor glycemic control (82.9%) than T2DM patients (57.5%) [16]. Additionally, a study showed that older patients and those with more than one complication or comorbidity experienced delays in achieving optimal glycemic control [17, 18]. Furthermore, research indicated that patients living in urban areas, with lower BMI levels, no family history of diabetes, higher education levels, abstaining from alcohol consumption, and using a combination of insulin and OHA treatment were more successful in controlling and reducing their blood sugar levels [19]. Recent evidence indicates that pharmacological interventions can improve glycemic control and may contribute to remission in diabetic patients [20, 21], highlighting the importance of integrating treatment strategies with clinical monitoring to enhance long‐term outcomes.

Recent studies have increasingly applied fractional‐order and fractal‐fractional models to better capture the nonlinear, memory‐dependent dynamics of glucose‐insulin interactions, improving understanding of system stability, bifurcation, and control strategies in diabetes management. However, these approaches are largely theoretical or simulation‐based, with limited application to real clinical data [22, 23, 24, 25, 26, 27]. In contrast, the present study applies a joint modeling framework for longitudinal and survival data, where log‐transformed blood sugar measurements are analyzed using a linear mixed‐effects model, providing a practical and clinically relevant approach to assessing disease progression and time to remission.

There have been numerous studies conducted on DM using the linear mixed model (LMM) for longitudinal outcomes and the Cox proportional hazards (Cox‐PH) model for time‐to‐event data. While these models are powerful tools on their own, analyzing them separately overlooks the intrinsic relationship that may exist between the longitudinal process (e.g., blood sugar levels over time) and the time‐to‐event outcome (e.g., time to first remission). Joint modeling offers a statistically rigorous framework that simultaneously analyzes both data types, allowing for the correlation between the longitudinal biomarker and the event process to be explicitly modeled [28, 29, 30, 31]. This integrated approach can yield more accurate and efficient estimates by borrowing strength across the two processes and accounting for shared individual‐specific random effects.

In the clinical context of diabetes, blood sugar levels are measured regularly, often every 3 months, and these repeated measures reflect disease progression and patient adherence to treatment. Fluctuations in blood sugar levels are clinically meaningful, as they may indicate an increased or decreased likelihood of remission or the onset of complications. By jointly modeling these longitudinal glucose measurements with the time to first remission, we can better understand how trends in blood sugar levels influence remission outcomes [30, 31]. This has direct clinical implications: such insights could aid in developing dynamic, individualized prognostic tools that support timely interventions, improving patient outcomes and resource allocation in diabetic care

Although the prevalence of diabetes mellitus is rising in Ethiopia and the study area, few studies have jointly analyzed longitudinal blood sugar level (BSL) and time to first remission. To address this gap, the present study applies a joint modeling approach to identify factors associated with both BSL trends and remission time among T1DM patients at Debre Tabor General Hospital (DTGH).

Operational Definition

**First Remission**: refers to the initial period when blood sugar levels return to a normal range and remain there for at least 3 months, without the need for glucose‐lowering medication. In clinical terms, remission is when your HbA1c, a measure of blood glucose level, remains below 48 mmol/ml or 6.5% for at least 3 months, without diabetes medication [32, 33, 34].

**Type I diabetes mellitus:** It is a chronic condition where the body's immune system mistakenly attacks and destroys the insulin‐producing cells in the pancreas. This leads to a deficiency in insulin, a hormone crucial for regulating blood sugar levels.

**Type II diabetes mellitus:** It is a chronic condition where the body either doesn't produce enough insulin or can't effectively use the insulin it produces, leading to high blood sugar levels. It's characterized by insulin resistance, where the body's cells don't respond properly to insulin, and a gradual decline in insulin production by the pancreas.

**Blood Sugar Level**: Normal blood sugar levels typically fall between 70 and 100 mg/dL when fasting (after not eating for at least 8 h) and below 140 mg/dL 2 h after eating. The expected values for normal fasting blood glucose/sugar concentration are between 70 mg/dL (3.9 mmol/L) and 100 mg/dL (5.6 mmol/L).

**Retrospective Study**: is a research design that analyzes data that has already been collected, examining past events and outcomes. Retrospective studies look/recall backward in time to investigate the relationship between exposures and outcomes that have already occurred.

## 2 Methods

### 2.1 Study Setting and Design Overview

The study was conducted at DTGH, Debre Tabor, Northwest Ethiopia. The area is located 667 km away from Addis Ababa, the capital city of the country. The hospital has specialty chronic illness clinics where patients with specific chronic diseases are referred for follow‐up. A retrospective study design was carried out to retrieve relevant information from the medical records of T1DM patients to address the objective of the study.

### 2.2 The Study Population and Study Period

The study population was T1DM patients who were under the follow‐up of insulin medication at DTGH and started medication in January 2018, up to January 2020.

### 2.3 Source of Data and Data Collection Procedures

Data for this study were collected from the medical charts of T1DM patients in the outpatient department (OPD) section at the hospital who were under follow‐up from January 2018 to January 2020. The data were collected by two statisticians and one medical doctor recruited from July 12, 2020, to July 27, 2020. The longitudinal and survival data would be extracted from the patient's chart, which contains socio‐demographic and clinical information of all T1DM patients under the follow‐up. The longitudinal response variable BSL measurements were measured in mg/dl, and the first remission from T1DM was extracted depending on the first time the patient was at a normal sugar level. The patient must complete at least three follow‐up visits within the study period to be eligible for remission. The longitudinal outcome variable, BSL, is measured approximately every 3 months, regardless of patient visits to the OPD for chronic disease management at DTGH. Patients have a total of nine visits: at baseline, and then at 3, 6, 9, 12, 15, 18, 21, and 24 months. The patient charts are prepared by the Federal Ministry of Health to be uniformly used by clinicians to early identify and document clinical and laboratory measurements. Thus, this study used secondary data obtained from patient follow‐up charts, and no need to get the study participants.

### 2.4 Inclusion‐Exclusion Criteria

**Figure 1:**
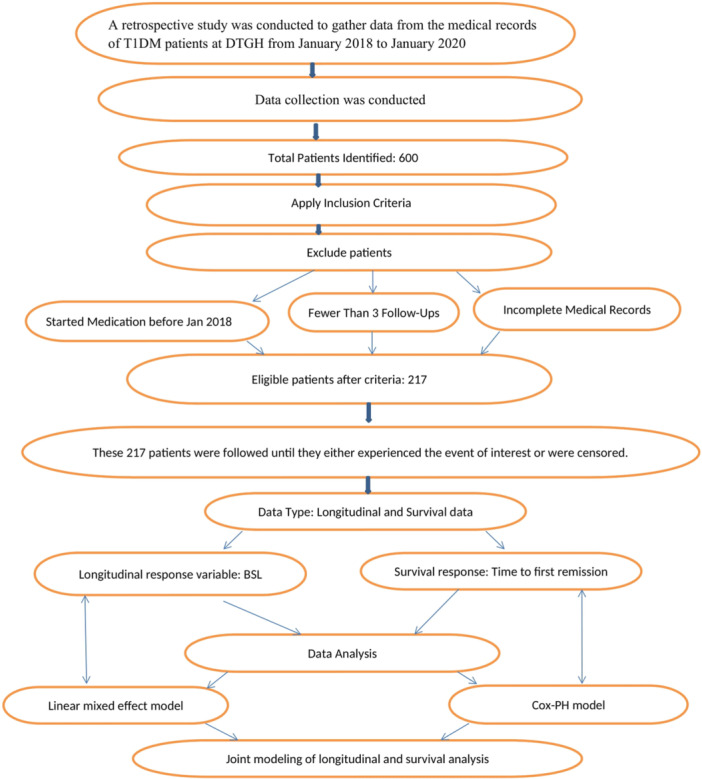
Flowchart of this retrospective study.

Participants included in this study were T1DM patients receiving insulin therapy at DTGH during the study period. Eligible individuals had attended at least three follow‐up visits, possessed complete medical records with the necessary information for analysis, and belonged to any age group. While Patients who started medication before the study period, patients who have been transferred or discharged from the treatment center before reaching the event of first remission, patients with fewer than three follow‐ups, and Patients with missing or incomplete medical records or information necessary for analysis were excluded from the study. These inclusion and exclusion criteria were designed to ensure the selection of a representative and reliable sample while minimizing the risk of bias or confounding in the study outcomes. The flowchart in Figure 1 visually outlines the participant selection process and the methodologies employed to analyze data from these participants.

Overall, a total of 600 T1DM patients were identified during the study period. Following the application of the inclusion and exclusion criteria, only 217 patients qualified for the study and were included in the final analysis. These patients were subsequently followed up until they either experienced the specified event or were censored.

### 2.5 Ethical Approval and Consent to Participate

Ethical approval for this study was obtained from Bahir Dar University Research and Ethics Committee with reference number PRCSVD/137/2020. As the study was based on secondary data and there was no direct contact with participants, the need for individual informed consent was waived by the committee.

### 2.6 Variables in the Study

**Response variables**: The two response variables in the current investigation were blood sugar level measured in mg/dl and time to first remission for T1DM patients (the time from the start of the treatment until it reaches the first normal blood sugar level in the follow‐up period).

### 2.7 Independent Variables

The study considers the following potential explanatory variables: Age in years, Gender (male, female), Place of residence (rural, urban), Presence of related disease (no, yes), Family history of diabetes mellitus (FHDM) (no, yes), Creatinine in MI/S, Weight in kg, and Hemoglobin in g/dL.

To minimize confounding, these covariates were selected based on clinical relevance and evidence from previous literature. In both the longitudinal and survival models, these variables were included as fixed effects to control for their influence on the outcomes. Multicollinearity was assessed using the variance inflation factor (VIF), and only non‐collinear predictors were retained. Additionally, in the joint modeling framework, the shared random effects allowed for simultaneous adjustment of confounders influencing both blood sugar progression and time to remission. This comprehensive modeling strategy improves the robustness and validity of our findings.

### 2.8 Data Analysis

Data were analyzed using SAS version 9.4 and R version 4.0.0. SAS was used for data management, descriptive statistics, and graphical exploration. Descriptive statistics were summarized using frequency tables and means with standard deviations for categorical and continuous variables, respectively. To assess normality assumptions, Q‐Q plots and individual profile plots of blood sugar level measurements.

The main statistical analyses were conducted in R. Survival experience across different groups of predictor variables was compared using the Log‐rank test, implemented using the survival package. The longitudinal data were analyzed using linear mixed‐effects models via the nlme package, while the Cox proportional hazards model was used for survival analysis. Joint modeling of longitudinal and time‐to‐event data was performed using the JM package.

### 2.9 Longitudinal Data Analysis

A key goal in statistical analysis is to understand and account for variability within data. In longitudinal studies, this variability arises from two primary sources: differences within individuals over time (within‐subject variation) and differences between individuals (between‐subject variation) [35, 36]. The linear mixed effects model is a commonly applied method for analyzing longitudinal data with continuous outcomes [36].

The Linear Mixed Effects Model incorporates both fixed and random components; the fixed effects capture the overall average response across the population, while the random effects account for individual‐specific variations. Let $y_{\mathrm{ij}}$ denote the blood sugar measurement for subject i at time $t_{\mathrm{ij}}$, where i=1,2,N and $j=1,2,n_{i}$. The vector of observations for subject i is given by $y_{i}={\left(y_{i1},y_{i2},y_{\mathrm{in}}\right)}^{T}$.

The linear mixed model proposed by Laird and Ware can be written as:

(3.1) $${\;y}_{i}=X_{i}\beta +Z_{i}b_{i}+{Є}_{i}$$

Where: $y_{i}$ is the nx1 vector of observed response values, β is the p x1 vector of fixed effect parameters,$\;X_{i}$ is the $n_{i}\mathrm{x p}$ observed design matrix of corresponding to the fixed effects, $b_{i}$ is the qx1 vector of random effect parameters for subject i, and $z_{i}$ is the $n_{i}\mathrm{xq}$ observed design matrix corresponds to the random effects, and ${Є}_{i}$ is the $n_{i}x\;1$ vector of residual errors. The corresponding assumption for the model (3.1) is $b_{i}\sim N(0,D$), and ${Є}_{i}\sim$N (0, Σ), where D and Σ denote the variance‐covariance matrices of the random effects and residual errors, respectively [37].

### 2.10 Survival Data Analysis

For the survival sub‐model, we used the Cox proportional hazards model, a semi‐parametric model appropriate for analyzing time‐to‐event data [38]. The hazard function for time to first remission was expressed as:

(3.2) $$h\left(t,x_{i},\beta \right)=h_{0}(t)\text{exp}(x_{i}^{T}\beta )$$

Where: $h_{0}(t)$ is the baseline hazard function; $x_{i}$ is a vector of covariates, and β is a vector of parameter estimates. Note that $h_{0}(t)$ is the hazard function, where all values of the covariates are zero ($\text{exp}(x_{i}^{T}\beta )=1$). Parameter estimate β refers to the increase in log hazard with a one‐unit increase for the continuous covariate. We checked the proportional hazards assumption using Schoenfeld residuals, and no significant violations were found [39].

### 2.11 Joint Modeling for Longitudinal and Survival Data

When longitudinal data is correlated with survival data, fitting separate models for each kind of data may not give complete information. To solve this problem, we consider incorporating the longitudinal measures directly into the Cox PH model as time‐varying covariates and then proceeding with the Cox proportional hazard model analysis [28, 30].

### 2.12 Longitudinal Sub‐Model

The longitudinal component of the joint model was specified using a linear mixed effects model. Let $y_{\mathrm{ij}}$ denote the blood sugar measurement for subject i at time $t_{\mathrm{ij}}$, where $j=1,2,,n_{i}$. The longitudinal sub‐model is defined as

(3.3) $$y_{i}\left(t\right)=x_{i}^{T}\left(t\right)\beta +z_{i}^{T}\left(t\right)b_{i}+\varepsilon_{i}\left(t\right)={\;m}_{i}\left(t\right)+\varepsilon_{i}\left(t\right)$$

Where

$$m_{i}\left(t\right)=\;x_{i}^{T}\left(t\right)\beta +z_{i}^{T}\left(t\right)b_{i}$$

represents the true underlying longitudinal trajectory. Here, $x_{i}\left(t\right)$ and $z_{i}\left(t\right)$ denote the design vectors for the fixed effects β, and random effects $b_{i}$ respectively. The random effects follow a multivariate normal distribution $b_{i}\sim N\left(0,G\right)$, and the error terms are assumed to follow a multivariate normal distribution $\varepsilon_{i}\left(t\right)\sim N(0,\sigma^{2})$. The error terms($\varepsilon_{i}\left(t\right)$) are assumed to be independent of the random effects [30].

### 2.13 Survival Sub‐Model

The linear mixed effects model that incorporates patient‐specific blood sugar level intercepts and slopes is used for the longitudinal sub‐model, while the survival model is used to describe the time‐to‐first remission of T1DM patients for the survival sub‐model. Then, the two sub‐models are linked through a shared parameter random effect [30]. Let ${T_{i}}^{*}$ and $C_{i}$ be the true event time and censoring time, respectively, for subject i. The observed event time is defined as $T_{i}=\min({T_{i}}^{*},C_{i})$, and the event indicator $\delta_{i}$ = *I* $\left({T_{i}}^{*}\leq C_{i}\right)$, which indicates the event indicator with $\delta_{i}=0$ when the event time is censored and $\delta_{i}=1\;$otherwise.

To examine the association between $m_{i}\left(t\right)$ and the hazard for an event, the survival sub‐model for Cox proportional hazard model is given by

$$h_{i}\left(\mathrm{t|}m_{i}\left(t\right),w_{i}\right)=\lim_{\delta t\to 0}\left\{\frac{P\left[t\leq T_{i}^{*}<t+\delta t,|,T_{i}^{*}\geq t,m_{i}\left(t\right),w_{i}\right]}{\delta t}\right\}$$

(3.4) $$h_{0}\left(t\right)\text{exp}\{\gamma^{\prime }w_{i}+\alpha m_{i}(t),\;t>0\;,$$

where, $m_{i}\left(t\right)=\left\{m_{i}\left(s\right),0\leq s<t\right\}$is the true values of longitudinal covariate up to time $t,h_{0}\left(t\right)$is the unspecified baseline hazard function or the hazard for a reference individual with all covariate values 0, $w_{i}$is the vector of the baseline covariate, andγ is the vector of regression coefficients corresponding to the vector $w_{i}$. The effect of the longitudinal outcome on the survival outcome at time t is measured by the parameterα The hazard model (34) assumes that the hazard for an event at time t depends only on the present values of the time‐varying covariate $m_{i}\left(t\right)$ [30].

The parameters of the joint model were estimated using the maximum likelihood approach via the Expectation–Maximization (EM) algorithm. The EM algorithm iteratively updates parameter estimates by alternating between the expectation (E‐step) and maximization (M‐step) until convergence is achieved. In this study, convergence was assessed based on the change in the log‐likelihood between successive iterations, with a predefined tolerance level of less than $1\times 10^{-6}$. This criterion ensures stability and reproducibility of the parameter estimates [40]. The structure of the joint log‐likelihood function is specified in the Supporting file Supp 1.

### 2.14 Model Selection

Model selection aimed to balance parsimony and fit. The best model was chosen based on: Akaike Information Criterion (AIC), standard errors of parameters, the significance of the association parameter (α), and the correlation between random slope and intercept [41].

### 2.15 Model Diagnostics and Validation

For the longitudinal sub‐model, two frequently used types of residuals, standardized marginal and standardized subject‐specific residuals, were used [42]. These residuals were plotted against time and fitted values to check for patterns indicative of model misfit or non‐linearity. Normal Q‐Q plots of residuals were also assessed to evaluate the assumption of normality. For the survival sub‐model, diagnostic checks included Schoenfeld residuals to test the proportional hazards assumption, and Cox‐Snell residuals were used to assess overall model fit [43]. These residuals were plotted against the Nelson‐Aalen cumulative hazard to confirm model adequacy.

For the joint model, convergence of the EM algorithm was monitored using log‐likelihood trace plots across iterations. The final joint model specification was validated using fivefold cross‐validation, where the dataset was partitioned into five subsets. The model was iteratively trained on four subsets and tested on the remaining one, ensuring that each subset served as a test set once. Predictive accuracy of the model was assessed using root mean square error (RMSE) for the longitudinal component and concordance index (C‐index) for the survival component.

## 3 Results

Descriptive statistics for the predictor variables are presented in Table 1. From a total of 217 DM patients, about 67.7% got the event of first remission, and 32.3% were censored. Among 217 T1DM patients included in the study, 53.5% were females and 52.1% were from urban areas. The mean and SD (Standard deviation) of age at the start of the treatment were 32.61 and 11.54, respectively. The rest of the variables were described in the same way.

**Table 1:** Summary statistics for independent variables included in the study.

Variable	Category	Observed events (%)	Censored (%)	Total (%)
Gender	Male	46 (21.2)	55 (25.3)	101 (46.5)
	Female	101 (46.5)	15 (6.9)	116 (53.5)
Residence	Urban	100 (46.1)	13 (6.0)	113 (52.1)
	Rural	47 (21.6)	57 (26.3)	104 (47.9)
FHDM	No	112 (51.6)	57 (26.3)	169 (77.9)
	Yes	35 (16.1)	13 (6.0)	48 (22.1)
Other related diseases	No	122 (56.2)	32 (14.8)	154 (71.0)
	Yes	25 (11.5)	38 (17.5)	63 (29.0)
Albumin	No	99 (45.6)	49 (22.6)	148 (68.2)
	Yes	48 (22.1)	21 (9.7)	69 (31.8)

**Baseline measured continuous covariates**		
**Covariate**	**Mean**	SD
weight	58.32	6.451
Age	32.61	11.54
hemoglobin	12.87	1.9241
Creatinine	0.9864	0.1418

### 3.1 Data Exploration for Longitudinal Data

**Figure 2:**
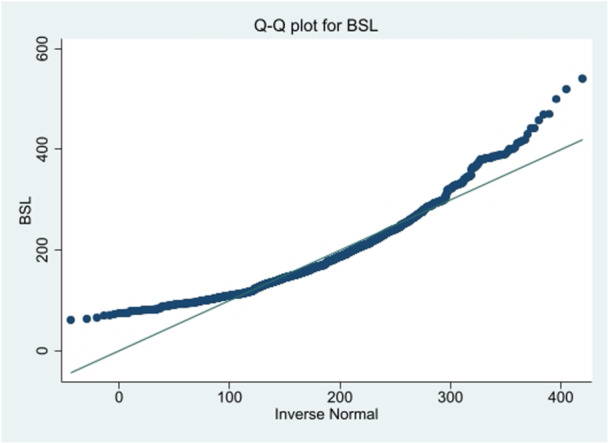
Q–Q plot for baseline blood sugar level (BSL).

**Figure 3:**
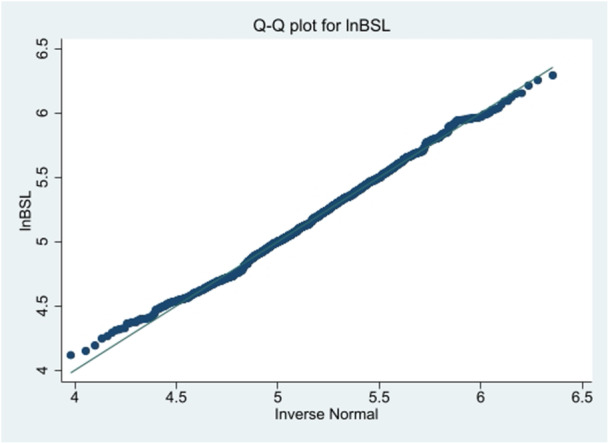
Q–Q plot of natural log‐transformed blood sugar level (lnBSL).

Figure 2 displays the Q‐Q plot for the BSL measurement of the original data. The Q‐Q plot for the original data was rightly skewed, as a result, some transformation must be made to meet the assumptions. Therefore, we used natural log transformation as natural log of blood sugar (lnBSL) shown in Figure 3 since it satisfies the available assumptions.

### 3.2 Profile Analysis

**Figure 4:**
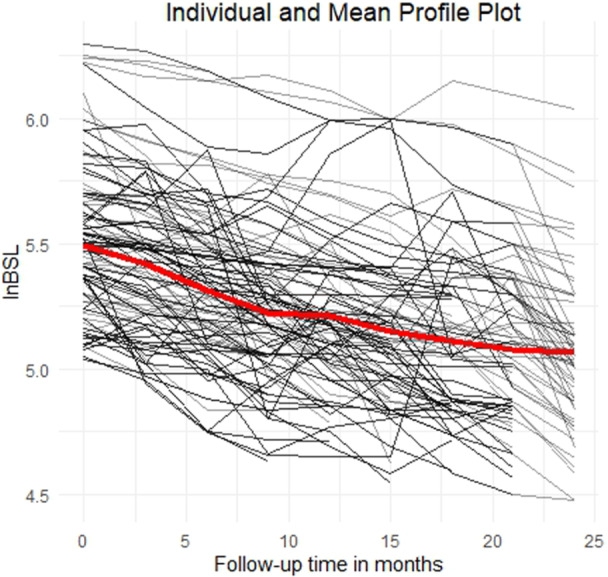
Individual and mean profile plot of lnBSL over time.

Figure 4 illustrates individual longitudinal trajectories and the mean profile of natural log‐transformed BSL (lnBSL) measurements over a 24‐month follow‐up period. Each thin black line represents an individual patient's lnBSL measurements, reflecting substantial within‐ and between‐subject variability throughout the observation period. Notably, the central red curve superimposed on the individual trajectories denotes the mean profile of lnBSL across all patients at each time point. This mean profile provides a smoothed representation of the central trend in lnBSL levels over time.

Clinically, the plot indicates that patients generally exhibited elevated lnBSL levels at baseline, which progressively declined following initiation of treatment. The downward trajectory of the red line suggests a consistent and sustained reduction in average lnBSL values over time, potentially signifying a favorable response to the therapeutic intervention. This pattern underscores the overall effectiveness of the treatment protocol in reducing blood sugar levels over time, while also highlighting the importance of accounting for individual variability in longitudinal assessments.

### 3.3 The Separate Longitudinal Analysis

The separate analysis belongs to each of the two responses namely BSL and time to recovery. For continuous BSL measurement, we used LMM (linear mixed model). Table 2 shows that the variables FHDM, gender, hemoglobin, another related disease, and visit time in months were significant at a 5% level of significance for the final linear mixed model. In addition, all random effect parameters showed statistically significant results.

**Table 2:** Result of the final linear mixed model for T1DM.

Covariate	Estimate	SE	95% CI		*p*‐value
			Lower	Upper	
Intercept	4.5059	0.1237	4.2632	4.7483	< 0.001***
Age	0.0029	0.0015	0.0002	0.0058	0.051
Gender (ref = female)					
Male	0.0732	0.0367	0.0013	0.1451	0.05*
Residence (rural)					
Urban	−0.0625	0.0517	−0.1639	0.0388	0.22
Related disease (ref = no)					
Yes	0.0683	0.0311	0.0073	0.1293	0.03*
FHDM (ref = no)					
Yes	0.0747	0.0364	0.0033	0.1461	0.04*
Hemoglobin	−0.0663	0.0217	−0.1088	−0.0239	0.003**
Creatinine	0.0271	0.0319	−0.0313	0.1312	0.39
Visit time	−0.0273	0.0014	−0.0304	−0.0246	< 0.001*

**Random effect**	SD	**95% CI**	
		Lower	upper
Intercept ($b_{0i}$)	0.1438	0.1108	0.1768
Visit time ($b_{1i}$)	0.0163	0.0142	0.0184
Corr $\left(b_{0i},b_{1i}\right)$	−0.5384	−0.6201	−0.4567
Residual ($\varepsilon_{i}$)	0.1341	0.0942	0.1745
**Selection Criteria**			
AIC	1057.4		
BIC	−008		

* *p* ≤ 0.05

** *p* ≤ 0.01

*** *p* ≤ 0.001.

### 3.4 The Separate Survival Analysis

Before fitting the Cox‐PH model, the proportional hazards assumption was tested using a global test. As shown in Table 3, all covariates have *p*‐values > 0.05, indicating no significant correlation between Schoenfeld residuals and survival time. The global test is also non‐significant (Chi‐Square = 6.92, *p*‐value = 0.33), confirming that the proportional hazards assumption is not violated. The separate survival analysis was conducted using the Cox proportional hazard model. Table 4, indicates that, among the potential predictors, age, gender, FHDM, and other related diseases were significantly associated with time to the first remission for T1DM patients at a 5% level of significance.

**Table 3:** Cox proportional hazard assumption test.

Variable	Chi‐square	df	*p*‐value
Age	1.85	1	0.17
Gender	2.31	1	0.13
Residence	0.95	1	0.33
FHDM	1.12	1	0.29
Related disease	0.88	1	0.35
Hemoglobin	2.76	1	0.09
**Global**	**6.92**	**6**	**0.33**

**Table 4:** The final Cox proportional hazards model.

Covariate	Estimate	SE	HR (95% CI)	*P*‐value
Age	−0.0232	0.0074	0.9770 (0.9628, 0.9914)	0.002*
FHDM (ref = no)				
Yes	−0.5077	0.1984	0.6018 (0.4079, 0.8880)	0.01*
Residence (ref = rural)				
Urban	−0.5931	0.2883	1.8096 (1.0284, 3.1843)	0.04*
Gender (ref = female)				
Male	−1.7501	0.2364	0.1737 (0.1093, 0.2762)	< 0.001***
Related disease (ref = no)				
Yes	−2.3390	0.4035	0.0964 (0.0437,0.2126)	< 0.001***
Hemoglobin	0.6907	0.3562	1.9952 (0.9925, 4.0110)	0.05*

* *p* ≤ 0.05

*** *p* ≤ 0.001.

### 3.5 Joint Model Analysis

Table 5 displayed the result of the joint model of longitudinal blood sugar levels and time to the first remission of T1DM patients. The association parameter between blood sugar levels and time to first remission was highly significant (∝ = −1.7914, *p*‐value < 0.001). This negative association indicates that higher blood sugar levels are associated with a longer time to reach the first remission. Specifically, a one‐unit increase in log‐transformed blood sugar level is associated with a reduction to 0.167 times the hazard of remission (exp(∝) ≈ 0.167), corresponding to an approximately 83.3% decrease in the hazard. In clinical terms, patients with elevated blood sugar levels are likely to experience delayed remission, highlighting the importance of tighter blood sugar control in promoting earlier remission in diabetic patients.

**Table 5:** Joint model analysis.

Covariate	Longitudinal sub model			Survival sub model			
	Estimate	SE	*p*‐value	Estimate	SE	HR	*p*‐value
Intercept	4.5624	0.1182	< 0.001***				
Age	0.0028	0.0012	0.02*	−0.0257	0.0072	0.9746	< 0.001***
Gender (ref=female)							
Male	−0.0727	0.0362	0.05*	−1.7683	0.2360	0.1706	< 0.001***
Residence (ref=rural)							
Urban	0.0621	0.0514	0.22	0.6716	0.2748	1.9574	0.01**
related disease (ref=no)							
Yes	0.0654	0.0308	0.03*	−2.5475	0.3827	0.0783	< 0.001***
FHDM (ref=no)							
Yes	0.0752	0.0341	0.03*	−0.5259	0.1976	−2.661	0.008**
Hemoglobin	−0.0659	0.0199	< 0.001***	0.7809	0.3293	2.1833	0.02*
Creatinine	0.0198	0.0327	0.56				
Visit time	−0.0281	0.0012	< 0.001***				
Assoc				−1.7914	0.2163	0.1668	< 0.001***

**Random effects**	**Std dev**	95% CI	
Intercept ($b_{0i}$)	0.1432	0.1342	0.1522
Visit time ($b_{1i}$)	0.0159	0.0152	0.0166
Corr $\left(b_{0i},b_{1i}\right)$	−0.5538	−0.5552	−0.5524
Residual ($\varepsilon_{i}$)	0.1327	0.1297	0.1357

**Selection Criteria**	
AIC	−1065.7
BIC	−1022.9

* *p* ≤ 0.05

** *p* ≤ 0.01

*** *p* ≤ 0.001.

Furthermore, variables age, gender, other related diseases, FHDM, hemoglobin, and visit time in months were statistically significant predictors of the longitudinal sub‐model, and variables age of patient, residence, FHDM, gender, other related diseases, and hemoglobin were significant predictors of time to first remission of T1DM patients.

The interpretation of the parameter in the longitudinal sub‐model and survival sub‐model was as follows: In the longitudinal sub‐model, the estimated coefficient of fixed effect intercept was 4.5624, which indicates that the average mean value of lnBSL level for patients was 4.5624 mg/dL, keeping the effect of other factors constant (*p*‐value < 0.001). For a unit increase in age, the average lnBSL of patients was significantly increased by 0.0028 mg/dL (*p*‐value = 0.02), keeping all other variables constant. For a unit increase in hemoglobin, the average lnBSL of patients was significantly decreased by 0.0659 mg/dL (*p*‐value < 0.001), keeping all other variables constant.

The average lnBSL of the patients who had other related diseases was significantly higher by 0.0654 mg/dL (*p*‐value = 0.03) compared to the patients who hadn't the disease, keeping other variables remaining constant. The average lnBSL of the patients with FHDM was significantly higher by 0.0752 mg/dL (*p*‐value = 0.03) compared to the patients with no FHDM, keeping other variables remaining constant. The average lnBSL of male patients was significantly higher by 0.0727 mg/dL (*p*‐value = 0.04) compared to the female patients keeping other variables constant. For a unit increase in visit time, the average lnBSL of T1DM patients was significantly decreased by 0.0281 mg/dL (*p*‐value < 0.001), keeping other variables constant.

According to the survival sub‐model, the rate of achieving the first remission for male patients was lower by 82.9% compared to female patients (HR = $e^{-1.7683}$ = 0.1706, *p*‐value < 0.001). That means the time needed to reach the first remission for male patients was significantly longer compared to female patients. The rate of achieving the first remission for patients with other related diseases was lower by 92.17% compared to patients with no other related disease (HR = $e^{-2.5475}$ = 0.0783, *p*‐value < 0.001). This means the time needed to reach the first remission among patients with other related diseases was significantly longer compared with patients with no other related disease.

Similarly, the rate of achieving the first remission for urban patients was 95.74% higher than for rural patients (HR = $e^{0.6716}$ = 1.9574, *p*‐value = 0.01). That means the time needed to reach first remission for urban patients was significantly shorter compared to rural patients.

For a unit increase in age, the rate of achieving first remission for patients was decreased by 2.5 3% (HR = $e^{-0.0257}$ = 0.9746, *p*‐value < 0.001). The estimate of the association parameter was ∝ = −1.7914 (*p*‐value = < 0.001), which shows that there was a strong negative association between the natural logarithm of fasting blood sugar measurement and time to first remission for T1DM patients.

The variance components from the joint model were explicitly examined to assess the partitioning of variability. The random effects covariance matrix (**G**) showed notable between‐subject variability, with standard deviations of 0.1432 for the random intercept and 0.0159 for the random slope, and a negative correlation (−0.5538) between them, indicating interdependence between baseline blood sugar levels and their rate of change over time. The residual variance (**Σ**), representing within‐subject variability, had a standard deviation of 0.1327.

To evaluate predictive performance in the joint model, fivefold cross‐validation was conducted. The longitudinal sub‐model demonstrated good predictive accuracy with a root mean square error (RMSE) of approximately 0.13, indicating a close agreement between observed and predicted log‐transformed blood sugar levels. For the survival sub‐model, the concordance index (C‐index) was approximately 0.76, suggesting good discriminative ability of the model in predicting time to first remission.

### 3.6 Sensitivity Analysis

To assess the robustness of the association parameter, a sensitivity analysis was conducted using alternative random‐effects structures in the longitudinal sub‐model. Specifically, a joint model with a random intercept only was compared to the primary model with both random intercept and random slope. The estimated association parameter remained negative and statistically significant across model specifications. In the primary model (random intercept and slope), the association parameter was estimated as ∝=−1.7914(SE=0.2163,p<0.001), while in the reduced model with random intercept only, the estimate was ∝=−1.7348(SE=0.2592,p<0.001). The similarity of these estimates indicates that the relationship between blood sugar level and time to first remission is robust to the specification of the random‐effects structure.

### 3.7 Comparison of the Separate and Joint Models

When comparing the results of the separate and joint models, the joint model (Table 5) demonstrated smaller standard errors than the separate models (Tables 2 and 4), indicating that it provides more precise parameter estimates. This improvement was further quantified using relative efficiency, where most parameters showed values greater than one, indicating increased precision under the joint modeling framework (Table 6). For instance, relative efficiency values of 1.56 for age and 1.36 for visit time in the longitudinal component highlight substantial gains in estimation efficiency. Similarly, in the survival component, relative efficiency values were generally greater than or equal to one, confirming comparable or improved precision of the joint model estimates. Beyond statistical precision, the joint model offers clinical relevance by simultaneously capturing the dynamic relationship between blood sugar level and time to first remission. This enables a more integrated understanding of disease progression, which is particularly valuable in clinical settings where multiple outcomes are interrelated. The joint model estimated a significant association parameter (∝ = −1.7914, *p*‐value < 0.001). providing strong evidence of dependency between the two sub‐models. In quantitative terms, a one‐unit increase in log‐transformed blood sugar level reduces the hazard of remission to approximately 0.167 times ($e^{-1.7914}$ ≈ 0.167), corresponding to an 83.3% decrease in the hazard, indicating substantially delayed remission among patients with higher blood sugar levels. This result implies that patients with higher blood sugar levels tend to experience delayed remission, a clinically important insight that would not be as clearly captured using separate models.

**Table 6:** Relative Efficiency Comparison between Separate and Joint Models.

Relative efficiency for (Separate longitudinal vs joint models)			
**Covariate**	**SE (Separate LMM)**	**SE (Joint)**	**Relative Efficiency**
Intercept	0.1237	0.1182	1.09
Age	0.0015	0.0012	1.56
Male	0.0367	0.0362	1.03
Urban	0.0517	0.0514	1.01
Related disease	0.0311	0.0308	1.02
FHDM	0.0364	0.0341	1.14
Hemoglobin	0.0217	0.0199	1.19
Creatinine	0.0319	0.0327	0.95
Visit time	0.0014	0.0012	1.36

Relative efficiency for (Separate survival vs joint models)			
**Covariate**	**SE (Separate Cox‐PH)**	**SE (Joint)**	**Relative Efficiency**
Age	0.0074	0.0072	1.06
FHDM	0.1984	0.1976	1.01
Urban	0.2883	0.2748	1.10
Male	0.2364	0.2360	1.00
Related disease	0.4035	0.3827	1.11
Hemoglobin	0.3562	0.3293	1.17

Moreover, the joint model showed smaller residual variability compared to the separate linear mixed‐effects model. This suggests a better fit and improved predictive accuracy, as the joint model more effectively accounts for the correlation between the two outcomes. Specifically, in the joint model, the variability in random intercepts (0.1432) exceeded that of random slopes (0.0159), and the correlation between intercepts and slopes was −0.5538, indicating a notable interdependence. The within‐patient variability (standard error) was also lower at 0.1327. Additionally, the correlation coefficient between random intercept and slope supports the notion that these parameters are not independent, reaffirming the need for a model that can accommodate such relationships. Furthermore, model selection criteria supported the superiority of the joint model, with lower AIC (−1065.7) and BIC (−1022.9) values compared to the separate longitudinal model (AIC = −1057.4, BIC = −1008.7), indicating improved overall model fit. In contrast to separate models that treat each outcome in isolation, the joint modeling framework provides a unified structure that enhances both interpretability and clinical applicability. Overall, in terms of variability, model fit, significance of association, and clinical interpretability, the joint model clearly outperformed the separate models and was thus preferred.

## 4 Discussion

The main objective of this study was to identify the factors that affect the longitudinal Blood Sugar measurement and time to first remission at Debre Tabor General Hospital, Debre Tabor, Ethiopia. This study employed three distinct analytical approaches: a linear mixed effects model to analyze the longitudinal lnBSL, a Cox proportional hazards model to assess the time to first remission independently, and a joint model to simultaneously examine both outcomes.

The study revealed that the average lnBSL increases with age, and as age increases, the time to achieve remission decreases. This result was consistent with another study [14, 17, 18]. In their finding, the time to reach optimal glycemic control for older patients was longer as compared to younger.

Hemoglobin (the measurement of anemia) was also another statistically significant variable for the longitudinal sub‐model; thus, as hemoglobin level increases, lnBSL decreases. This result was consistent with another study [15], which showed that among T1DM patients with a similar level of glycemia, iron deficiency anemia was associated with higher concentrations of HbA_1c_. This result was also consistent with [44]. In their finding, Anemia is an independent risk factor for CVD outcomes in subjects between the ages of 45 and 64 years.

The average lnBSL was found to evolve differently between patients with other related diseases and patients without other related diseases based on the results of separate and joint models. The average lnBSL was higher for patients who had other related diseases compared to patients who had no other related disease. Also, Patients who had other related diseases prolonged their first remission time. This result was consistent with another study [18]. This result was also consistent with another study [14]. In their findings, patients with additional complications have poor glycemic control.

Urban patients in our study achieved remission in a shorter time compared to their rural counterparts. This aligns with previous research [19], which indicated that urban residents were better equipped to manage and reduce blood sugar levels over time, possibly due to greater access to healthcare facilities, better health literacy, and more structured lifestyles. However, this finding is contradicted by other studies [45, 46], which reported that urban dwellers exhibited a higher likelihood of metabolic risk factors. This discrepancy may stem not only from differences in methodologies, sample sizes, and data collection techniques, but also from sociocultural and lifestyle variations. For instance, urban environments may promote sedentary behavior and unhealthy diets, which can elevate metabolic risks despite better healthcare access. Conversely, rural patients may benefit from more physically active lifestyles but have limited access to diabetes education and treatment facilities.

Regarding gender differences, our study found that male T1DM patients had higher average lnBSL levels, and females achieved first remission more quickly. This finding is consistent with previous research [17, 47, 48], which suggests that males tend to recover more slowly than females. The contradiction found in other studies [49], where females showed higher HbA1c levels, could be attributed to population‐specific physiological or behavioral factors. For example, the above study was conducted in Germany, and differences in healthcare systems, lifestyle habits, or even hormonal and immunological profiles may contribute to these contrasting results. In the Ethiopian context, it is possible that female patients are more compliant with treatment regimens or benefit from stronger familial and social support systems, potentially facilitating earlier remission.

Family history of diabetes mellitus (FHDM) also played a significant role in our study. Patients with a family history of diabetes had higher average lnBSL levels, suggesting a genetic predisposition to poorer glycemic control. This finding supports the study by [19], which emphasized that individuals without a FHDM may find it easier to monitor and regulate their blood sugar levels. However, other studies have reported that family history does not significantly influence treatment outcomes [50], while yet another study [51] highlighted the genetic interplay between type 1 and type 2 diabetes, asserting that family history can shape the phenotype and disease progression. These inconsistencies could be explained by the multifactorial nature of diabetes. The interplay of genetic inheritance, environmental exposure, and lifestyle factors complicates the relationship between FHDM and diabetes management. Moreover, the perception of risk due to family history might affect patient behavior differently in various cultural or socioeconomic contexts; some patients may become more vigilant, while others might feel fatalistic or less motivated to change.

This study demonstrated a statistically significant negative association between blood sugar levels and time to remission. Specifically, a one‐unit increase in log‐transformed blood sugar level was associated with approximately 83.3% reduction in the hazard. This indicates that patients with higher blood sugar levels tend to experience slower or delayed remission. Clinically, this finding underscores the importance of effective glycemic control, as reducing blood sugar levels may substantially improve the likelihood and timing of remission among diabetic patients. The statistical significance of ∝ this suggests that modeling these two processes jointly provided a better fit to the data compared to analyzing them separately. The joint modeling approach effectively captured the dependency between the progression of blood sugar levels over time and the timing of remission, which would have been overlooked in separate models. By accounting for the inherent correlation between the longitudinal and survival outcomes, the joint model improves estimation efficiency and reduces bias in the effect estimates. This finding is consistent with previous studies [52, 53], which also reported that the significant association parameter supports the superiority of joint modeling over separate analyses in capturing the interconnected nature of longitudinal and time‐to‐event data.

## 5 Conclusion

This study demonstrated that the joint modeling approach, which simultaneously analyzes longitudinal and survival data, provided a better fit compared to separate survival and longitudinal models. Specifically, the parameter estimate for the unobserved true lnBSL was ∝ = −1.7914, $e^{-1.7914}=0.167$, indicating a significant negative association between blood sugar levels and time to first remission. This suggests that as blood sugar levels decrease, remission is achieved more quickly. The joint model exhibited superior performance in terms of goodness of fit, reduced variability, and statistically significant association parameters.

Clinically, the findings highlight the need for targeted interventions for high‐risk groups; namely, older patients, males, individuals with comorbid conditions, those with a family history of diabetes mellitus, and patients with anemia, as these groups demonstrated higher blood sugar levels and longer times to remission. Health professionals and policymakers should prioritize comprehensive and continuous care strategies for these populations, including early screening, routine monitoring, and tailored health education to manage disease progression more effectively.

From a methodological standpoint, this study supports the application of joint models in evaluating both longitudinal biomarker trajectories and clinical endpoints. Future research should consider extending the joint modeling framework to broader diabetic populations and to include additional clinical outcomes like the development of complications or treatment adherence. Moreover, incorporating time‐varying covariates and exploring interaction effects over time would enhance the model's robustness and relevance.

### 5.1 Limitations of the Study

One of the primary limitations of the study was the model's inability to account for interaction effects of predictors over time due to convergence issues arising from variable patient visit times. Additionally, there was limited literature focused on T1DM in the study area, as most existing research in Ethiopia centers on T2DM, making comparative analysis challenging. Another limitation was the absence of critical predictor variables such as body mass index (BMI), feeding style, and physical activity from patient records. The exclusion of these variables may have introduced residual confounding, potentially biasing the estimates of predictor‐outcome relationships. For instance, BMI and physical activity are well‐established factors influencing both glycemic control and remission outcomes in diabetic patients. Furthermore, the retrospective design may introduce potential biases, such as inaccuracies or inconsistencies in medical record documentation, which could affect the reliability of the data.

To address these limitations, future studies should aim to include more comprehensive clinical and behavioral data, possibly through robust data collection or improved electronic health record systems. In addition, further research should be conducted on T1DM in underrepresented settings, using larger and more diverse samples, to develop a more holistic understanding of its determinants and progression.

## Author Contributions

**Maru Zewdu Kassie:** conceptualization, investigation, writing – original draft, methodology, validation, visualization, writing – review and editing, software, formal analysis, project administration, data curation, resources. **Awoke Seyoum Tegegne:** investigation, writing – review and editing, visualization, methodology, validation, project administration, supervision, data curation, software, resources.

## Funding

The authors have nothing to report.

## Disclosure

All authors have read and approved the final version of the manuscript. The corresponding author had full access to all data in this study and takes complete responsibility for the integrity and accuracy of the data analysis. The corresponding author (Maru Zewdu Kassie) also affirms that this manuscript is an honest, accurate, and transparent account of the study being reported, that no important aspects have been omitted, and that any discrepancies from the planned study (and, if relevant, registered) have been explained.

## Conflicts of Interest

The authors declare no conflicts of interest.

## Use of Artificial Intelligence (AI)

No AI tools were used in the generation of scientific content. AI‐assisted tools were used only for language editing.

## Transparency Statement

The lead author, Maru Zewdu Kassie, affirms that this manuscript is an honest, accurate, and transparent account of the study being reported; that no important aspects of the study have been omitted; and that any discrepancies from the study as planned (and, if relevant, registered) have been explained.

## Supporting information


**Supporting File:** hsr272427‐sup‐0001‐Supplemetary_material.docx.

## Data Availability

The data that support the findings of this study are available on request from the corresponding author. The data are not publicly available due to privacy or ethical restrictions. The datasets generated and/or analyzed during the current study are available from the corresponding author upon reasonable request.
