# Supplementary material for: Robustness of Joint Over Separate Models for Investigating Predictors of Blood Sugar Level and Time to First Remission Among Type I Diabetic Patients Under Treatment; a Retrospective Study Design
Source: Health Sci Rep. 2026 Apr 24;9(5):e72427. doi: 10.1002/hsr2.72427 (PMC13109078; doi:10.1002/hsr2.72427)
Supplement: Supplementary file 1 — Supporting File: hsr272427‐sup‐0001‐Supplemetary_material.docx. [file HSR2-9-e72427-s001.docx]

**Joint Log-Likelihood Function**

The joint model for longitudinal and survival data is based on the joint likelihood of the repeated measurements and time-to-event outcomes. Let $y_{i}$ denote the longitudinal measurements and ($T_{i}, \delta_{i}$) the survival outcome for subject $i$. The joint likelihood is given by:

$$L\left( \theta\right)=\prod_{i=1}^{n} \int f(y_{i}/b_{i};\theta)f(T_{i},\delta_{i}/b_{i};\theta)f(b_{i};\theta)db_{i}$$

Where $b_{i}$ represents the subject-specific random effects, and $\theta$ denotes the vector of model parameters. The corresponding log-likelihood function is:

$$l\left( \theta\right)=\sum_{i=1}^{n} \log\int f(y_{i}/b_{i};\theta)f(T_{i},\delta_{i}/b_{i};\theta)f(b_{i};\theta)db_{i}$$

The integral over the random effects doesn’t have a closed-form solution and is approximated using numerical techniques within the EM algorithm.

**Supplementary Table 1:** Log rank tests for categorical variables

| **Variable** | **Chi-square** | **df** | **p-value** |
| --- | --- | --- | --- |
| Gender | 28.5 | 1 | <0.001 |
| Related disease | 32.8 | 1 | <0.001 |
| FHDM | 6.5 | 1 | 0.01 |
| Residence | 4.2 | 1 | 0.04 |
